# e-Cadherin in 1-Methyl-4-phenyl-1,2,3,6-tetrahydropyridine-Induced Parkinson Disease

**DOI:** 10.1155/2016/3937057

**Published:** 2016-04-17

**Authors:** Samuela Cataldi, Michela Codini, Stéphane Hunot, François-Pierre Légeron, Ivana Ferri, Paola Siccu, Angelo Sidoni, Francesco Saverio Ambesi-Impiombato, Tommaso Beccari, Francesco Curcio, Elisabetta Albi

**Affiliations:** ^1^Department of Pharmaceutical Science, University of Perugia, 06100 Perugia, Italy; ^2^Inserm U 1127, CNRS UMR 7225, Sorbonne Universités, UPMC Université Paris 06 UMR S 1127, Institut du Cerveau et de la Moelle ÉPINIÈRE, ICM, 75013 Paris, France; ^3^Institute of Pathologic Anatomy and Histology, University of Perugia, 06100 Perugia, Italy; ^4^Department of Clinical and Biological Sciences, University of Udine, 33100 Udine, Italy

## Abstract

Today a large number of studies are focused on clarifying the complexity and diversity of the pathogenetic mechanisms inducing Parkinson disease. We used 1-methyl-4-phenyl-1,2,3,6-tetrahydropyridine (MPTP), a neurotoxin that induces Parkinson disease, to evaluate the change of midbrain structure and the behavior of the anti-inflammatory factor e-cadherin, interleukin-6, tyrosine hydroxylase, phosphatase and tensin homolog, and caveolin-1. The results showed a strong expression of e-cadherin, variation of length and thickness of the heavy neurofilaments, increase of interleukin-6, and reduction of tyrosine hydroxylase known to be expression of dopamine cell loss, reduction of phosphatase and tensin homolog described to impair responses to dopamine, and reduction of caveolin-1 known to be expression of epithelial-mesenchymal transition and fibrosis. The possibility that the overexpression of the e-cadherin might be implicated in the anti-inflammatory reaction to MPTP treatment by influencing the behavior of the other analyzed molecules is discussed.

## 1. Introduction

1-Methyl-4-phenyl-1,2,3,6-tetrahydropyridine (MPTP) is a lipophilic nontoxic substance that crosses blood-brain barrier and accumulates in astrocytes where it is metabolized into the toxic metabolite, 1-methyl-4-phenylpyridinium (MPP+) by the monoamine oxidase-B (MAO-B) enzyme. MPP+ is released from astrocytes and transported into dopaminergic neurons where it acts as a potent inhibitor of mitochondrial complex I, compromising ATP synthesis and leading to activation of cell death pathway [1]. For this reason, MPTP was considered a neurotoxin and it was widely used for producing animal models of Parkinson disease (PD) resulting, primarily, in motor clinical symptoms as testified by resting tremors, rigidity, bradykinesia, and postural instability [2]. In PD chronic model, animals were injected with 10 doses of MPTP over 5 weeks at 3- and 5-day intervals and showed a significant reduction in the number of neurons in the substantia nigra pars compacta (SNpc) and striatal dopamine levels [3]. The effect depended on the dose per day, the duration, and the route of administration [4]. In PD acute or subacute models, animals were injected with MPTP through a number of different routes using various dosing regimens for the development of several distinct versions of the acute model (a low dose, an intermediate dose, 1-2 daily injections, and others) [4]. Mice treated for acute model showed less than half of the destroyed SNpc dopaminergic neurons and less reduction of nigrostriatal degeneration in comparison with chronic MPTP model [5]. Acute or subacute injections of MPTP resulted in a significant but reversible loss of dopaminergic functions while in chronic model alterations were final [6]. In PD acute model, early gene expression differed from that induced by PD chronic; changes of early genes were crucial for dopamine neuron death [7].

Inflammation had an important role in the pathogenesis of PD [8]. Dopamine loss correlated with a strong inflammatory response characterized by reactive microglia with large cell bodies and short processes [5]. Activated microglial cells and T lymphocytes were detected in the SNpc of PD patients concomitant with an increased expression of proinflammatory mediators [9]. Hsp60 released by injured neurons can specifically bind to microglial cells and stimulates the production of proinflammatory cytokines [10]. Moreover, activated astrocytes and microglial cells synthesize chemokines involved in brain defense mechanisms [11]. On the other hand, glial cells could release deleterious compounds such as proinflammatory cytokines (TNF-alpha, Il-1 beta, and IFN-gamma), which might be involved in apoptosis [12]. MPTP-induced microglial activation was characterized by the upregulation of IL-1*β* in the SNpc and of caspase-11 in the ventral midbrain in acute MPTP-treated mice [7]. Interleukin- (IL-) 1 beta, IL-2, IL-4, IL-6, and transforming growth factor-alpha levels were elevated in ventricular cerebrospinal fluid in PD patients [13]. Neuroinflammatory mechanisms might contribute to the cascade of events leading to neuronal degeneration. As part of these mechanisms, adaptive immune response to neurodegeneration has also been involved in neuronal cell death in PD. In particular, T cell-mediated dopaminergic toxicity was shown to be almost exclusively arbitrated by CD4+ T cells and required the expression of FasL but not IFN-gamma [13].

Cadherins are a family of transmembranous glycoproteins responsible for calcium-dependent cell-cell adherence, reduction of cell-cell recognition and sorting, coordination of multicell movements, and maintenance of cell and tissue polarity [14]. Cadherins were specifically expressed in the nervous system and had important roles in brain function and development [14]. Neural cadherin (n-cadherin) and epithelial cadherin (e-cadherin) belong to the same family of type I. n-cadherin regulated neuronal polarity and neuron development [14] whereas e-cadherin, known to be a tumor suppressor, played a role in meningioma [15]. While the protective effect of n-cadherin on dopaminergic neuron [16] and its implication on neurodegeneration [17] had been demonstrated, so far no information exists about the behavior of e-cadherin in PD.

We aimed to study the behavior of e-cadherin in the acute MTPT model of PD in relation to molecules involved in neuroinflammation and neurodegeneration such as tyrosine hydroxylase (TH), interleukin-6 (IL-6), phosphatase and tensin homolog (PTEN), and caveolin-1.

## 2. Methods

### 2.1. Animals

Ten- to twelve-week-old male C57BL/6 J mice, weighing 25–30 g (CERJ, France), were used. Mice were kept in a temperature-controlled room (23°C ± 1°C) under a 12-hour light/dark cycle and had* ad libitum* access to food and water. Animal handling was carried out according to ethical regulations and guidelines (Guide for the Care and Use of Laboratory Animals; NIH publication number 85-23; revised 1985) and the European Communities Council Directive 86/609/EEC. Experimental protocols were performed following the French national chart for ethics of animal experiments (articles R 214-87 to 126 of the “Code rural”) and received approval from the ethical committee number 5 “Charles Darwin” and from the ICM animal care and use committee.

### 2.2. Reagents

Anti-e-cadherin, anti-TH, anti-PTEN, and anti-caveolin-1 antibodies and secondary antibodies for immunoblotting and immunofluorescence were obtained from Santa Cruz Biotechnology, Inc. (California, USA); anti-IL-6 was obtained from Thermo Fisher Scientific, Waltham, Massachusetts, USA; anti-neurofilament (NF) 200 kDa antibodies were from NOVOCASTRA Laboratories Ltd. (Newcastle, UK).

### 2.3. MPTP Injection and Tissue Preparation

Groups of mice (*n* = 5) received MPTP under an acute protocol. Mice were given 4 i.p. injections of MPTP-HCl (Sigma) 2 hours apart and at a dose of 20 mg/kg (free-base). They were euthanized 7 days after the last MPTP injection. Control mice received an equivalent volume of 0.9% NaCl solution. Removed brains were postfixed overnight in fresh 4% paraformaldehyde (PFA)/phosphate-buffered saline (PBS) solution, cryoprotected with 30% sucrose in 0.1 M PB, and frozen in isopentane (−30°C). Brain free-floating sections (20 *μ*m thick) encompassing the entire midbrain were prepared using a freezing microtome (Microm, Germany). Three different sections, representative of three different rostrocaudal levels of the midbrain, were used. The sections were dropped with specific orientation in paraffin. The paraffin blocks were sectioned into 4 *μ*m thick sections. All sections were mounted on silan-coated glass slides and used for morphological, immunohistochemical, and immunofluorescence analysis. Samples of three different sections (20 *μ*m thick) were collected and used for immunoblotting analysis.

### 2.4. Morphological Analysis

The sections were deparaffinized, rehydrated through a series of xylene and ethanol washes, and analyzed by phase contrast microscopy EUROMEX FE 2935 (ED Amhem, The Netherlands) equipped with a CMEX 5000 camera system. The analysis of the tissue section size was performed by ImageFocus software, as previously reported [18].

### 2.5. Immunohistochemistry

Bond Dewax solution was used for removal of paraffin from tissue sections before rehydration and immunostaining on the Bond automated system (Leica Biosystems Newcastle Ltd., UK) as previously reported [19]. Immunostaining for NF200 detection was performed by using specific antibodies and Bond Polymer Refine Detection, Leica Biosystems (Newcastle Ltd., UK) [19]. The observations were performed by using inverted microscopy EUROMEX FE 2935 (ED Amhem, The Netherlands) equipped with a CMEX 5000 camera system.

### 2.6. Western Immunoblotting

The proteins were quantified as previously reported [18]. About 30 *μ*g of pellet proteins was submitted to SDS-PAGE electrophoresis in 10% polyacrylamide slab gel for e-cadherin, TH, and PTEN and 12% slab gel for IL-6 and caveolin-1 detection. Electrophoresis image analysis was performed on gels stained with Coomassie-Blue. Proteins were transferred into nitrocellulose for 90 min as previously described [20]. The membranes were blocked for 30 min with 0.5% no-fat dry milk in PBS (pH 7.5) and incubated overnight at 4°C with the specific antibody. The blots were treated with HRP-conjugated secondary antibodies for 90 min. Visualization was performed with the enhanced chemiluminescence kit. The apparent molecular weight of the proteins was calculated according to the migration of molecular size standards. The area density of the bands was evaluated by densitometry scanning and analyzed with Scion Image [20].

### 2.7. Immunofluorescence Analysis

After deparaffinization, rehydration through a series of xylene and ethanol washes, and 3 washes with phosphate-buffered saline, sections were incubated with 2 *μ*g/mL anti-e-cadherin and anti-TH primary antibodies diluted in a 0.5% solution of bovine serum albumin in PBS overnight at 4°C. The slides were washed 3 times with PBS and incubated for 1 hour at room temperature with fluorescein isothiocyanate (FITC) secondary antibodies. After 3 washes with PBS, the slides were mounted with glycerol and coverslips. The samples were examined under a fluorescence microscope (Zeiss Axiophot) equipped with an Olympus DP 50 camera system.

### 2.8. Statistical Analysis

Three experiments were performed for each analysis. Data are expressed as mean ± SD and *t*-test was used for statistical analysis between control and experimental samples.

## 3. Results and Discussion

### 3.1. Results

**Figure 1:**
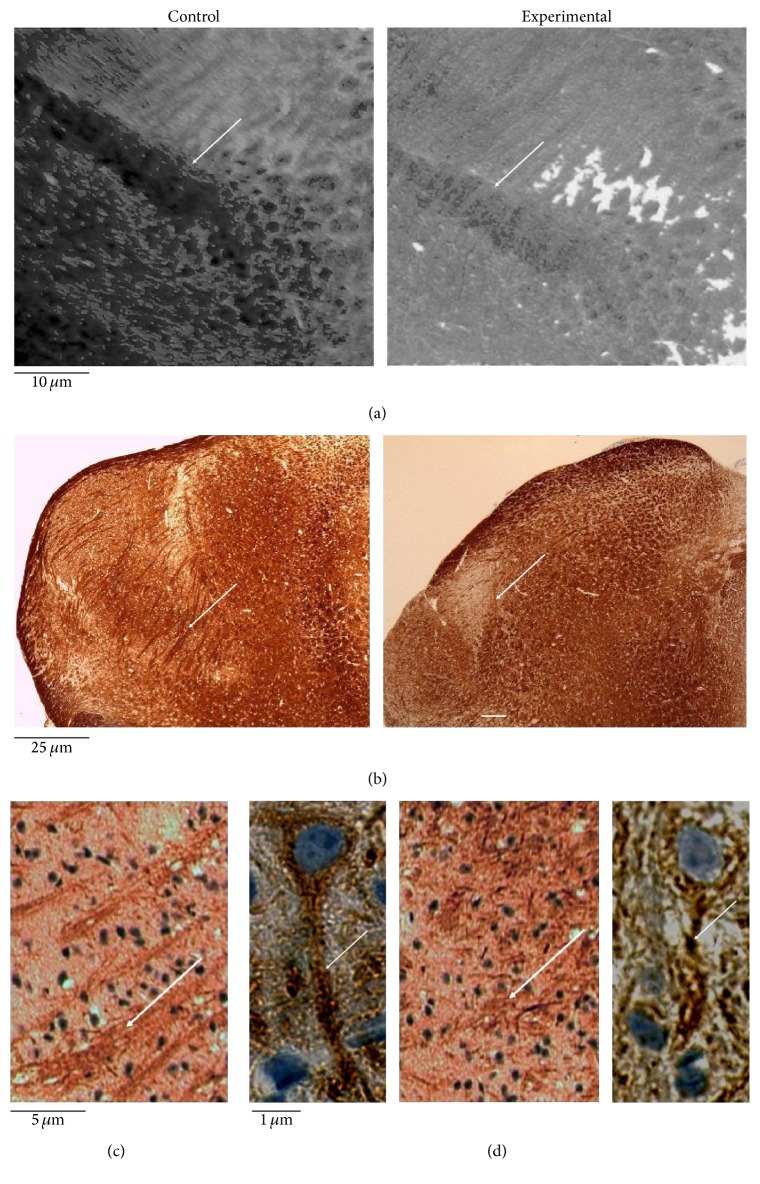
Midbrain of untreated (control) or MPTP-treated (experimental) mice. The samples were treated as reported in Section 2. (a) Phase contrast images of substantia nigra, 10x magnification; (b) neurofilament 200 kDa (NF200) immunohistochemical staining, 4x magnification, (c) 20x magnification, and (d) 100x magnification. The arrows indicate the effect of MPTP treatment in the reduction of area with neurofilaments (a) and of length and thickness of neurofilaments (b, c, d).

In order to study the role of e-cadherin in neuroinflammation associated with dopaminergic injury, we first investigated the impact of MPTP toxicity on midbrain structure. Microscopy analysis, performed on histological microsections, confirmed a loss of SN surface area (Figure 1(a)), widely reported in literature [3–5]. The analysis of heavy neurofilament (200–220 kDa) by immunohistochemistry showed that MPTP treatment reduced the length and thickness of neurofilaments (Figure 1(b)). At higher magnification, alteration of the axon structure in MPTP-treated animals compared to controls was evident (Figure 1(c)).

**Figure 2:**
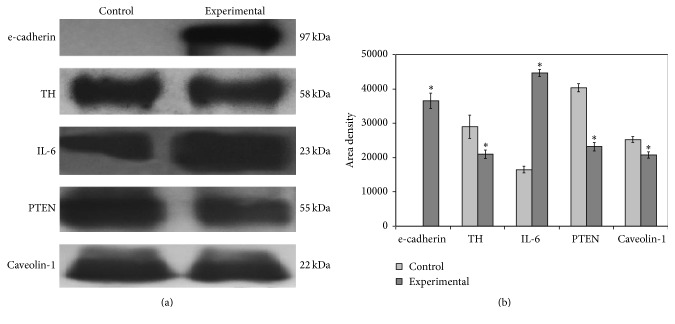
(a) Immunoblotting analysis of e-cadherin, tyrosine hydroxylase (TH), interleukin-6 (IL-6), PTEN, and caveolin-1. The position of the proteins was evaluated in relation to that of molecular size standards. (b) The area density was quantified by densitometry scanning and analysis with Scion Image; the data represent the mean ± SD of three separated experiments. ^*∗*^ *P* < 0.001 versus control sample.

**Figure 3:**
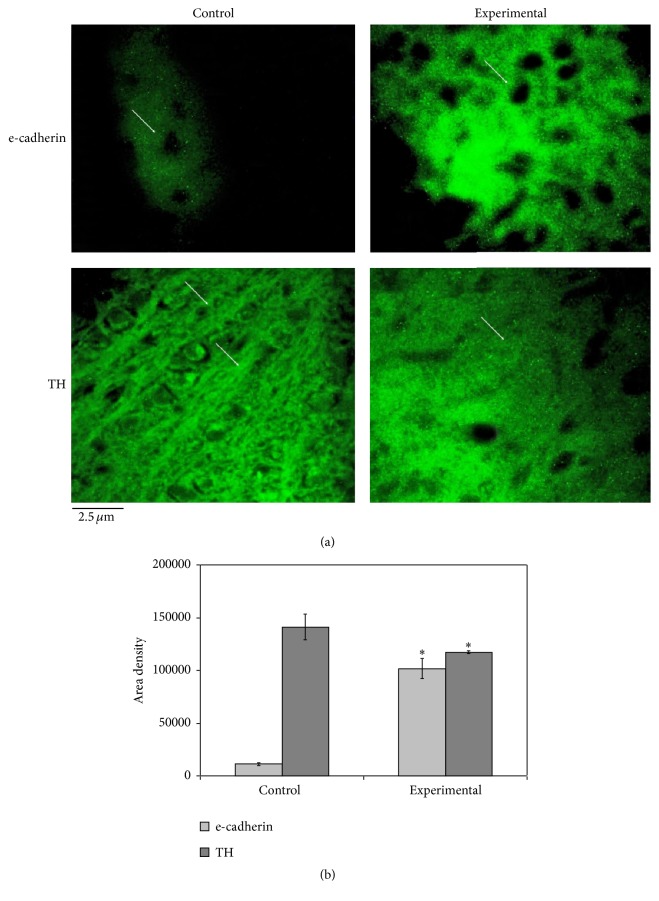
(a) Fluorescence immunostaining of e-cadherin and tyrosine hydroxylase (TH) by using specific antibodies in the substantia nigra area of control and experimental midbrain. 40x magnification. (b) The immunofluorescence area density was quantified by densitometry scanning and analysis with Scion Image; the data represent the mean ± SD of three separated experiments. ^*∗*^ *P* < 0.001 versus control sample. The arrows indicate the distribution of e-cadherin and TH fluorescence; e-cadherin is randomly distributed in control sample and has specific localization around the soma in experimental sample. TH florescence is evident around the neurons and along the neurofilaments in control sample whereas it is distributed uniformly in the tissue surrounding the cells.

Then, we asked whether the structural modifications of neurons observed after MPTP treatment could be related to their functional changes. To address this question, immunoblotting analysis of midbrain tissue homogenates was performed using specific antibodies against different markers. Our results show the following: (1) a reduction of TH levels in MPTP-treated mice compared to control mice as expected from previous reports [21, 22]; (2) an increase of the IL-6 signal (band corresponding to a 23 kDa apparent molecular weight) and decrease of the PTEN and caveolin-1 signals (bands corresponding to 55 kDa and 22 kDa apparent molecular weight, resp.) (Figure 2(a)). Quantification of band area densities demonstrated that IL-6 increased 2.7 times whereas TH, PTEN, and caveolin-1 decreased 1.38, 1.74, and 1.21, respectively (Figure 2(b)). Surprisingly, e-cadherin was undetectable in protein homogenates from saline-treated mice and showed remarkable upregulation in diseased animals (MPTP-treated mice) (Figures 2(a) and 2(b)). Immunofluorescence analysis was performed by focusing the attention on the SN area. As expected from the immunoblotting data, the immunofluorescence signal for e-cadherin was very low in control samples and strongly increased in experimental samples, with specific localization around the soma (Figure 3(a)). TH was reduced in experimental sample, supporting immunoblotting results, and had a different distribution (Figure 3(b)). Indeed, in control sample the florescence was evident around the neurons and along the neurofilaments whereas in experimental sample it was distributed uniformly in the tissue surrounding the cells (Figure 3(b)) therefore supporting the immunohistochemical study showing structural changes of the neurofilaments (Figures 1(c) and 1(d)).

### 3.2. Discussion

Administration of MPTP has had dangerous effects on dopaminergic neurons of the nigrostriatal pathway in animals to produce experimental model of parkinsonism [1–4] with neuroinflammatory mechanisms [23]. IL-1*β*, IL-2, IL-4, and IL-6 were elevated in ventricular cerebrospinal fluid in juvenile parkinsonism and PD [7]. IL-6 immunoreactivity was observed within microglia and astrocytes during MPTP-induced dopamine loss [24]. Here we showed a strong increase of IL-6 in the midbrain of MPTP-treated mice indicating that neuroinflammatory processes are brought into play and may be involved in the damage of neurofilaments. e-cadherin overexpression in Raw 264.7 macrophages inhibited their inflammatory response to LPS stimulation, with reduction of IL-6 release [25]. On the other hand, IL-6 reduced the expression of e-cadherin [26]. Such negative feedback between e-cadherin and IL-6 is, for still unknown reasons, inefficient during neuroinflammation-associated dopaminergic injury. Indeed, while e-cadherin expression was strongly stimulated after MPTP-induced injury, IL-6 levels were also found highly induced. This is the first observation of the behavior of the e-cadherin reported in the literature. Considering the anti-inflammatory effect of e-cadherin [25], we can speculate however that the overexpression of e-cadherin following nigrostriatal pathway injury could limit the inflammatory culprits and therefore reduce non-cell autonomous mechanisms of dopaminergic cell death. Based on this hypothesis, IL-6 expression, neurofilament damage, and reduction in TH and PTEN expression could be even stronger in the absence of e-cadherin. The reduction of TH is associated with dopamine cell loss [21] and inhibition of striatal PTEN in Parkinsonian rats resulted in motor dysfunction and impaired responses to dopamine [27]. The limitation of the inflammatory reaction might be also due to the reduction of caveolin-1. Caveolins are the main structural components of caveolae, plasma membrane invaginations implicated in different cell functions. In particular, caveolin-1 was the most expressed one in the membranes, and its genetic loss in mice resulted in almost complete loss of caveolae* in vivo*, the absence of caveolin-1 in mice leading to increased epithelial-mesenchymal transition and fibrosis [28]. The reduction of caveolin-1, by stimulating mesenchymal cells, could be a response to the MPTP-induced damage of midbrain structure that we showed. This hypothesis could be supported considering that mesenchymal stem cells had protective effect in neurodegeneration of PD model mice [29].

## 4. Conclusions

In conclusion, we showed for the first time an overexpression of e-cadherin in the midbrain of PD mice which could be a limiting factor to the neuroinflammatory damage. It will be interesting in the future to perform experiments in e-cadherin deficiency conditions to confirm this hypothesis and testify whether e-cadherin could represent a new therapeutic avenue for PD.
